# The right to health as the basis for universal health coverage: A cross-national analysis of national medicines policies of 71 countries

**DOI:** 10.1371/journal.pone.0215577

**Published:** 2019-06-28

**Authors:** S. Katrina Perehudoff, Nikita V. Alexandrov, Hans V. Hogerzeil

**Affiliations:** Global Health Unit, Department of Health Sciences, University Medical Centre Groningen, University of Groningen, Groningen, the Netherlands; University of Brescia, ITALY

## Abstract

Persistent barriers to universal access to medicines are limited social protection in the event of illness, inadequate financing for essential medicines, frequent stock-outs in the public sector, and high prices in the private sector. We argue that greater coherence between human rights law, national medicines policies, and universal health coverage schemes can address these barriers. We present a cross-national content analysis of national medicines policies from 71 countries published between 1990–2016. The World Health Organization’s (WHO) 2001 guidelines for developing and implementing a national medicines policy and all 71 national medicines policies were assessed on 12 principles, linking a health systems approach to essential medicines with international human rights law for medicines affordability and financing for vulnerable groups. National medicines policies most frequently contain measures for medicines selection and efficient spending/cost-effectiveness. Four principles (legal right to health; government financing; efficient spending; and financial protection of vulnerable populations) are significantly stronger in national medicines policies published after 2004 than before. Six principles have remained weak or absent: pooling user contributions, international cooperation, and four principles for good governance. Overall, South Africa (1996), Indonesia and South Sudan (2006), Philippines (2011–2016), Malaysia (2012), Somalia (2013), Afghanistan (2014), and Uganda (2015) include the most relevant texts and can be used as models for other settings. We conclude that WHO’s 2001 guidelines have guided the content and language of many subsequent national medicines policies. WHO and national policy makers can use these principles and the practical examples identified in our study to further align national medicines policies with human rights law and with Target 3.8 for universal access to essential medicines in the Sustainable Development Goals.

## Introduction

Universal access to essential medicines is an important component of the right to health and the Sustainable Development Goals (SDGs). [1] Essential medicines are those required to meet the priority health care needs of a population. [2] Realising universal access to medicines requires a coherent approach to medicines as essential public goods. [3]

The World Health Organization (WHO) advocates for the adoption of a national medicines policy (NMP) as a commitment to a goal and a guide to action. WHO’s 2001 guidelines to *Develop and implement a national drug policy* elucidate the key components of a NMP. [4] NMPs should be based on universal principles, involve a range of national stakeholders, and be tailored to the local context. [4,5] The first NMPs were predominantly adopted by low- and lower-middle income countries, and only since 2007 have many higher income countries followed. [5,6] By 2015, over 90% of low and middle-income countries (LMICs) had published a NMP. [6,7] Adopting a NMP has been associated with the provision of a basic range of essential medicines free at the point of care, and better quality use of medicines, particularly in LMICs. [8] Some NMPs are associated with improved antibiotic use in low-resource settings. [9] However, despite the development of NMPs in many countries, essential medicines have often remained inaccessible to many, especially vulnerable populations.

### Barriers to universal access

Most studies frame medicines as a single ‘input’ or commodity to be supplied in the health system. [3] This view leads to fragmented policies and interventions that fail to address the system-wide constraints (such as health care financing and medicine pricing) and consequently, have a limited effect on medicines access for vulnerable groups. [3] Moreover, public policy in many countries does not consistently recognise essential medicines as essential public goods, nor is medicines accessibility seen as part of the progressive realisation of the right to health. [10] This failure, among others, may be linked to stagnating public financing for medicines, insufficient financial protection for patients, high medicines prices, and a general indifference towards medicines inequities. [3,6,7,11,12] In response to these challenges, WHO promotes its policies for essential medicines and health systems as tools to design and assess comprehensive national strategies to achieve equitable and sustainable access to medicines. [2,4,13,14]

Public health policies and practices have changed since the turn of the century, which put much more emphasis on human rights and promoting universal health coverage (UHC). We therefore assert that greater coherence is now needed between NMPs’ goals and strategies, the wider health system including UHC, and human rights; and that incorporating human rights principles and the wider health systems perspective in NMPs can also create a supportive environment for medicines affordability and financing for vulnerable groups.

The right to health emanates from international treaties, most notably the 1966 International Covenant on Economic, Social, and Cultural Rights (ICESCR), which is ratified by 165 States. [15] These governments therefore bear the irrevocable duty to protect and promote the right to health. General Comment No. 14 (2000), an authoritative interpretation of the right to health by the UN Committee on Economic, Social, and Cultural Rights, establishes that governments have the ‘core obligation’ to provide essential medicines and to establish a national health strategy and plan of action. [16] Given that a NMP is a country-wide strategy for the pharmaceutical sector for the provision of essential medicines, State parties can be understood as having a legal obligation to establish and implement a NMP or similar policy. The right to health establishes universal minimum entitlements to essential medicines for all, a set of State duties and guiding principles for government action (i.e. transparency and participation), and mechanisms for rights enforcement and redress.

### Gaps in existing evidence

Despite the breadth of WHO’s 2001 guidelines, little evidence exists about integrating essential medicines components and right to health commitments in existing NMPs. [4] The largest cross-national comparison of NMPs examines their effectiveness and uptake in 64 mostly LMICs, using national indicators for quality use of medicines. [8] Other analyses are single-country or regional studies comparing NMPs against WHO’s essential medicines policies without large-scale cross-national comparisons. [17–19] No earlier studies have assessed the presence of right to health principles in NMPs.

An updated online repository of NMPs has only recently been established. NMPs are generally published in English or the national language, and were only available in hard copy or on local websites. Earlier policy studies therefore mostly relied on governments’ self-reports in the WHO Pharmaceutical Sector questionnaire of having a NMP or of its contents with binary (yes/no) answers. [8,18] In 2016 the Lancet Commission on Essential Medicines Policies undertook a first global search for all NMPs and later made them available to the WHO Essential Medicines Portal, which now provides easy access to these primary sources. [6]

Our article presents a first cross-national content analysis of essential medicines and right to health principles for access to medicines in 71 NMPs. Our comparison of NMPs also identifies some examples of strong text in support of universal access to medicines. These examples can inform policy makers who are developing and revising NMPs in the era of UHC and a strengthened right to health.

## Materials and methods

A detailed description of the methodology is in the online S1 Appendix.

### Data collection

Between January to October 2015 we conducted a systematic search of all NMPs mentioned in academic literature, published in online repositories and on government websites, and further expanded through a global call through the E-DRUG online network and targeted individual approaches. We included one official NMP per country. We excluded draft, incomplete, and unclear medicine policy documents, policies addressing a specific component (i.e. intellectual property management), and documents in other languages besides English, Dutch, French, or Spanish. This method, previously reported in Wirtz et al., yielded 67 full text NMPs, which were deposited in WHO’s online Essential Medicines and Health Products Information Portal (publicly accessible here: http://apps.who.int/medicinedocs/en/). [5] Between January 2017 to March 2018 we were able to identify 13 additional full text NMPs that met the inclusion criteria.

We recorded the year of the country’s most recent official NMP and its World Bank income category in the year of publication.

### Policy checklist

To analyse and assess the content of the NMPs, we developed a policy checklist by extracting the relevant principles for medicines affordability and financing from WHO’s policies for essential medicines, and from international human rights law. [2,4,13,16,20–22] These human rights documents were chosen because they list the provision of essential medicines as a core obligation or elaborate on the nature of State duties.

The policy checklist identifies 12 specific attributes of policy text for access to medicines and rates their strength on a 3-point scale (see Table 1). We (SKP, NVA) identified the 12 principles by first selecting a short list of concepts related to medicines affordability and financing from the above documents. We categorised the 12 principles into three domains (see Table 1 and the online S1 Appendix for a full description of the domains and principles): legal rights and obligations (i.e. government’s commitments and duties), good governance (i.e. governance principles and processes), and technical implementation (i.e. policy measures to achieve government objectives). The domains correspond to the structure-process-outcome framework for monitoring and evaluating the realisation of human rights. [23]

**Table 1:** Policy checklist for access to medicines in NMPs.

Checklist	Human rights principle	WHO essential medicines policy
**Legal rights and obligations**		
1. Right to health	Right to the highest attainable standard of health	Human rights are a ‘value’. [2]
2. State obligation to provide essential medicines	Core obligation to provide essential medicines defined by WHO	
**Good governance**		
3. Transparency	Transparency	Includes information to assess service access and coverage, and publicly available price information for medicines. [1,3] Also a component of good governance for medicines. [12]
4. Participation & consultation	Participation	Collaboration and accountability of all health systems actors, and stakeholder consultation. [1,3] Also vaguely referenced in good governance for medicines. [12]
5. Monitoring & evaluation	Monitoring	Achieved through explicit government commitment, indicator-based surveys, and independent impact evaluation. [1,3] Also a component of good governance for medicines. [12]
6. Accountability & redress	Accountability	Accountability of all health systems actors. [1] Also vaguely referenced in good governance for medicines. [12]
**Technical implementation**		
7. Selection of essential medicines	(Assured) quality of health services (of the AAAQ)	Includes the essential drugs concept, procedures to define and update the national list(s) of essential drugs, explicit, evidence-based criteria that includes cost-effectiveness, and selection mechanisms. [3,11]
	Duty to adopt appropriate legislative, administrative, budgetary and other measures to a maximum of its available resources Core obligation to provide essential medicines as defined by WHO	
8. Government financing		Requires adequate funding and mobilising all available public resources and increase funding for priority diseases, and the vulnerable. [1,3,11]
9. Pool user contributions		Medicines reimbursement with user charges is a (temporary) financing option. [1,11]
10. International assistance and technical cooperation	Duty to seek international assistance and technical cooperation	Includes the possibility of using development loans for medicines financing. [11]
11. Efficient and cost-effective spending	Duty for the efficient use of available resources Duty to take appropriate steps to ensure that the private business sector is aware of, and consider the importance of, the right to health in pursuing their activities. Duty to prevent unreasonably high costs for access to essential medicines from undermining the rights of large segments of the population to health. Duty to seek low-cost policy options.	Includes the efficient use of resources and affordable pricing through: price control; a pricing policy for all medicines; competition through generic policies and substitution; good procurement practices; price negotiation and information; and TRIPs-compliant measures such as compulsory licensing and parallel imports. [1,3,11]
12. Financial protection of vulnerable groups	Duty towards non-discrimination and attention to the vulnerable	Increase government funding for poor and vulnerable groups and reduce the risk of catastrophic health spending. [1,11]

Abbreviations used in this table: WHO = World Health Organization; TRIPs = Trade Related Aspects of Intellectual Property; AAAQ = Availability, Accessibility, Acceptability, and Quality as elements of health services under the right to health.

Through multiple, iterative rounds, we (SKP, NVA) independently piloted the short list on three NMPs and devised a 3-point coding matrix (see online S1 Appendix). After each round we revised the principles and coding matrix through consensus. The resulting framework was reviewed by three experts on the right to health and pharmaceutical policy (BT, HVH, EtH) for applicability to NMPs and accuracy of the definitions.

We calculated the reliability of NMP text selection by two coders using Cohen’s Kappa. SKP and NVA each independently coded six randomly selected countries (approx. 8% of the sample countries: Botswana, Ethiopia, Fiji, Malawi, Oman, Timor-Leste). We extracted the same NMP texts in 75.7% of cases with a Cohen’s K = 0.695, which indicates that 69.5% of similarities between coders were not due to chance (0.61<Cohen’s K<0.8 suggests ‘substantial’ agreement).

### Data analysis

Given the substantial agreement between coders, we (SKP, NVA) worked with one half of the NMPs each to extract the relevant text (through keyword and manual search) and coded the strength of each principle in the NMPs on a three-point coding matrix (i.e. strong, weak, or absent text, defined in the online S1 Appendix). Generally, strong text includes a clear State commitment to a principle and an action (i.e. to adhere to the concept of essential medicines and introduce a national selection committee) and where possible related to medicines affordability and financing. Weak text includes vague commitments. All text selections and codes were independently reviewed (by both SKP and NVA) who discussed any inconsistencies and jointly agreed on the final codes. We report the frequency of each principle in NMPs and describe the different approaches towards each principle in different countries.We hypothesised that the content of WHO’s 2001 NMP guidelines would inform the content of subsequent NMPs. Therefore we divided NMPs between those adopted in or before 2003 (n = 32) and those adopted in or after 2004 (n = 39). Associations were determined in SPSS version 25 using Pearson’s Chi-squared statistic with significance set at p<0.05.

## Results

Of the 80 full text NMPs we intitally retrieved, nine were excluded due to language restrictions or incompleteness. We included 71 NMPs published between 1990 and 2016. Our sample has a higher proportion of NMPs published before 2004 (≤2003 n = 32/47, 68% vs. ≥2004 n = 39/88, 44%) and by low income countries (n = 35/46, 76%) than middle and high income nations (n = 35/132, 27%).

The essential medicines and human rights principles included in each NMP are presented in Table 2. No NMP includes all of the 12 principles. NMPs with examples of innovative ideas are listed in Table 3 and the full text of these examples is available in the online S2 Appendix. The following sub-sections highlight the most relevant descriptive data for each principle.

**Table 2:** Overview of the 12 principles for access to medicines in NMPs from 71 countries.

NMP publisher	Date of publication	1. Right to health	2. State obligation	3. Transparency	4. Participation & consultation	5. Monitoring & evaluation	6. Accountability & redress	7. Selection of essential medicines	8. Government financing	9. Pool user contributions	10. International assistance & cooperation	11. Efficient & cost-effective spending	12. Financial protection of vulnerable groups
**WHO**	1988												
**WHO**	2001												
**Afghanistan**	2014												
**Albania**	1991												
**Andorra**	1999												
**Australia**	2000												
**Bangladesh**	2005												
**Barbados**	1999												
**Benin**	2008												
**Bhutan**	2007												
**Bolivia**	2003												
**Botswana**	2002												
**Burkina Faso**	1996												
**Cambodia**	2010												
**Central African Republic**	1995												
**Chad**	1998												
**Chile**	1996												
**Colombia**	2012												
**Comoros**	1997												
**Congo**	2004												
**Cote d’ivoire**	2009												
**Democratic Republic of Congo**	2002												
**Ecuador**	2007												
**El Salvador**	2011–2014												
**Eritrea**	2010												
**Ethiopia**	1993												
**Fiji**	2013												
**Finland**	2011												
**Gabon**	1999												
**Gambia**	1994												
**Ghana**	2004												
**Guinea**	1994												
**Haiti**	2014												
**Indonesia**	2006												
**Iran**	2004												
**Iraq**	2005												
**Jordan**	2014												
**Kenya**	2008												
**Kyrgyzstan**	2014												
**Liberia**	2001												
**Malawi**	1990–1995												
**Malaysia**	2012												
**Maldives**	2007												
**Mali**	2000												
**Mauritania**	2002												
**Namibia**	1998												
**Nepal**	1995												
**New Zealand**	2007												
**Niger**	1995												
**Nigeria**	2005												
**Oman**	2000												
**Pakistan**	1997												
**Peru**	2004												
**Philippines**	2011–2016												
**Rwanda**	2016												
**Senegal**	2006												
**Seychelles**	2009												
**Somalia**	2013												
**South Africa**	1996												
**South Sudan**	2006												
**Sri Lanka**	2006												
**Sudan**	2005–2009												
**Suriname**	2005–2008												
**Swaziland**	2011												
**Syria**	1992												
**Tajikistan**	2003												
**Tanzania**	1991												
**Timor-Leste**	2010												
**Togo**	1997												
**Trinidad and Tobago**	1998												
**Uganda**	2015												
**Vietnam**	1996												
**Zimbabwe**	2011												

Legend: Black = Strong text, Grey = Weak text, White = No text.

**Table 3:** Innovative NMP text for access to medicines.

**1. Right to health including essential medicines**
El Salvador (2011)—Southern Sudan (2006)
**2. State duty to provide pharmaceuticals**
Indonesia (2006)—Iran (2004)—Philippines (2011–2016)—Uganda (2015)
**3. Transparency of governments’ action and outcomes for medicines affordability**
Iran (2004)—Philippines (2011–2016)
**4. Participation and consultation for medicines affordability**
New Zealand (2007)
**5. Monitoring and evaluation for medicines affordability**
Colombia (2012)—Philippines (2011–2016)—Tajikistan (2003)
**6. Accountability and redress for medicines affordability**
Afghanistan (2014)—Kenya (2008)—Malaysia (2012)
**7. Selection of essential medicines**
Philippines (2011)—South Africa (1996)
**8. Sufficient government financing for essential medicines**
Afghanistan (2014)—Nigeria (2005)
**9. Pooling user contributions for essential medicines**
Eritrea (2010)
**10. International assistance and technical cooperations for medicines affordability**
Ecuador (2007)—Ghana (2004)
**11. Efficient and cost-effective spending on essential medicines**
Ecuador (2007)
**12. Financial protection of the poor and vulnerable**
Jordan (2014)—Philippines (2011–2016)—Timor Leste (2010)

### 12 principles for access to medicines in NMPs

#### 1. Right to health

Eleven NMPs frame access to medicines as part of the right to health (Congo 2004, Bhutan 2007, Kenya 2008, Colombia 2012, El Salvador 2011–2014, Kyrgyzstan 2014, Uganda 2015, Philippines 2011–2016, Rwanda 2016) and/or a right that governments must ensure (South Sudan 2006, Seychelles 2009). Kenya (2008) references the ICESCR and Colombia (2012) cites General Comment No. 14.

#### 2. State obligation

Access to medicines as a State obligation is mentioned in Syria (1992), Tajikistan (2003), Iran (2004), Indonesia and South Sudan (2006), El Salvador (2011–2014), Kyrgyzstan (2014), and the Philippines (2011–2016). Uganda (2015) requires the government to progressively realise UHC with essential services. Four NMPs (Congo 2004, Maldives 2007, Suriname 2005–2008, Timor Leste 2010) frame the government as being responsible for continuous medicines availability at an affordable price. The State must ensure the availability of medicines for all in need (South Africa 1996). Bhutan (2007) and Sudan (2005–2009) require the State to establish mechanisms to guarantee access for all to the medicines they need at an affordable price.

#### 3. Transparency

Eighteen NMPs mention the principle of transparency in relation to medicines prices, cost, or affordability. Notable examples reinforce the transparency of medicines selection and procurement (Malaysia 2012), funding decisions (New Zealand, 2007), pricing (Philippines 2011–2016), price information sharing including with the public (Philippines, Malaysia) and through a price database (Malaysia).

#### 4. Participation and consultation

South Africa (1996) and New Zealand (2007) include public participation in matters of medicines pricing and affordability.

#### 5. Monitoring and evaluation

Monitoring medicines prices serves to compare and widen tenders (Oman 2000), benchmark for setting domestic prices (Iran 2004), contain price increases (Malaysia 2012), to monitor affordability (South Sudan 2006), cost-efficiency and acceptability (Afghanistan 2014), or to determine the effects of international trade agreements on domestic access to medicines (Nigeria 2005). Monitoring is done by the Pricing Committee (Somalia 2013) or through a database (South Africa 1996) or an electronic essential medicines monitoring system (Philippines 2011–2016). Uganda (2015) frames monitoring progress towards equity and efficiency as part of the progressive realisation of the right to health. Tajikistan (2003) presents a robust list of indicators and Barbados (1999) adopts the indicators of the Harvard Drug Policy Research Group and Management Sciences for Health.

#### 6. Accountability

No NMP describes accountability in relation to medicines affordability or financing. The general principle of accountability is applied to medicines procurement (Pakistan 1997, Bhutan 2007), distribution (Botswana 2002), and financial management (Seychelles 2009, Swaziland 2011). Specific accountability mechanisms for general medicines issues are recognised in Kenya (2008), Malaysia (2012), and the Philippines (2011–2016).

#### 7. Medicines selection

Three NMPs indicate that the national essential medicines list (EML) serves as a basis for UHC (Uganda 2015) or reimbursement (Namibia 1998, Philippines 2011–2016). Frequent references are to the selection procedure (i.e. committee composition, periodicity of list, n = 40), the selection criteria (n = 27), or to the concept of essential medicines and/or the WHO Model List of Essential Medicines (n = 20). Less frequent was an explanation of the use or the purpose of an EML within the national health system (n = 15). Comprehensive NMPs that address multiple aspects of the selection of essential medicines are South Africa (1996), Pakistan (1997), Namibia (1998), Oman (2000), Nigeria and Iraq (2005), Maldives (2007), Malaysia (2012), Somalia (2013), and El Salvador (2011–2014).

#### 8. Government financing

Frequent references to government financing are for the provision of sufficient or adequate funding (n = 14) and to base medicines procurement and provision on objective health needs (n = 13). Less frequent is the duty of governments to dedicate funding to priority populations, priority diseases, or essential medicines (n = 7), to increase funding for medicines (n = 6) or to find alternate funding sources (n = 4).

Only Guinea (1994) and Indonesia (2006) set a quantitative threshold for government financing. In Guinea, the government spending target is US$ 0.25/inhabitant/year to finance ‘social medicines’ such as vaccines, anti-leprosy medicines and tuberculosis medicines. In Indonesia, a financing target must be set considering WHO’s then recommended minimum allocation of US$ 2.00/capita.

#### 9. Pooling user contributions

No NMP includes the principle of universal financial protection for users nor the compulsory pre-payment of contributions (usually through health insurance). The most comprehensive language is from South Africa (1996), Eritrea (2010) and Somalia (2013), which provide for free or low-cost access to medicines in primary care, and user contributions to finance medicines in secondary and tertiary care, with exceptions for people unable to pay. Botswana (2002) and Fiji (2013) adopt these principles as well, but without mentioning specific levels of care. A long-term objective in some NMPs was to develop health insurance and medicines reimbursement, e.g. in Namibia (1998), Tajikistan (2003), and Sri Lanka (2006).

#### 10. International assistance and technical cooperation

Ten NMPs describe assistance from the international community to promote the affordability of medicines. Assistance takes the form of technical cooperation and partnership for medicines accessibility (Malaysia 2012); bilateral and multilateral aid for essential medicines programmes (Gabon 1999, Democratic Republic of Congo 2002, Congo 2004); the financing for the public sector (Ghana 2004); mobilising resources for new essential medicines (Afghanistan 2014); reference pricing policies and price information exchange (Ecuador 2007); the negotiation of prices at sub-regional level (ANDEAN) and the exchange of information to prevent monopolistic practices (Peru 2004); to establish a donor coordination mechanism to document the finances used in procurement (Swaziland 2011). Colombia (2012) calls for the development of an interagency agenda for ‘*health diplomacy and access to medicines*’ that would include a National Health Technology Assessment to exchange methods, information, and capacities with national and international networks of experts.

#### 11. Efficient spending

Many NMPs describe various policy measures to achieve generic promotion (n = 37), pricing policies (n = 30), the use of flexibilities to Agreement on Trade-Related Aspects of Intellectual Property (TRIPS) and other measures to manage intellectual property (n = 21), tax exemptions (n = 16), pooled procurement (n = 8), price transparency (n = 7), and price negotiation (n = 7). NMPs that apply multiple, complementary policy measures are South Africa (1996), Ghana and Iran (2004), Nigeria (2005), Indonesia and South Sudan (2006), Ecuador (2007), the Seychelles (2009), Cambodia (2010), El Salvador (2011–2014), Jordan (2014), and the Philippines (2011–2016).

#### 12. Protection for the poor and vulnerable

Eighteen NMPs refer to medicines affordability or financing for specific populations such as children, people in remote or mountainous locations, ethnic groups, women, the disabled, or people with ‘priority diseases’ defined as tuberculosis, HIV, or malaria. Nine NMPs refer to medicines provision for general ‘vulnerable’ groups.

#### Trend analysis

Compared to WHO’s 1988 guidelines, the 2001 policy guidelines introduce strong commitments to individual rights, transparency, and measures for efficiency and cost-effectiveness, for pooling user contributions, for international cooperation, and for financial protection of the poor and vulnerable. Recommendations for medicines selection and government financing are strong in both WHO’s 1988 and 2001 guidelines.

Several trends are visible between NMPs published before or after 2004, including strong commitments to individual rights (≤2003: n = 0/32 (0%) vs. ≥2004: n = 13/39 (33%), p = 0.000), measures for government financed-medicines (6/32 (19%) vs 18/39 (46%), p = 0.015), for efficiency and cost-effectiveness (14/32 (44%) vs 29/39 (74%), p = 0.009), and for financial protection of the poor and vulnerable (4/32 (13%) vs 13/39 (33%), p = 0.041).

## Discussion

This paper presents a first cross-national comparison of the most recent NMPs from 71 countries, published between 1990–2016, using a 12-point checklist for universal access to medicines based on human rights and health system principles. The selection of essential medicines and their cost-effectiveness are the most frequent policy measures in our sample of NMPs. Good governance (transparency, participation, monitoring, or accountability for medicines affordability and financing), and measures to pool user contributions and to seek international cooperation remain weak or absent. An individual right to health, and measures for government financing of essential medicines, cost-effective spending, and financial protection of vulnerable groups are significantly stronger in NMPs published after 2004 than in those published before. NMPs with the clearest and strongest commitments to essential medicines and human rights principles are from South Africa (1996), Indonesia and South Sudan (2006), Malaysia (2012), Somalia (2013), Afghanistan (2014), Uganda (2015), and the Philippines (2011–2016); these texts may serve as models for others.

### Historical trends

Our findings suggest that some aspects of WHO’s 2001 guidelines were instructive and impactful on national pharmaceutical policy processes. Strong principles introduced in WHO’s 2001 guidelines are significantly more frequent in NMPs adopted in 2004 or later (i.e. an individual right to health, measures for cost-effective spending, and financial protection of vulnerable groups). Transparency is also significantly more common (although not always in relation to medicines affordability and financing) in NMPs adopted after 2003. Some important principles in WHO’s 2001 guidelines, such as measures to pool user contributions and to seek international cooperation, have rarely been taken up by any country. We cannot draw firm conclusions about the causal relation between WHO’s 2001 guidelines on NMP content, and subsequent national policies. We examined only the most recent NMP per country and cannot discount the possibility that certain countries already embraced the 12 principles in previous NMPs. Paired examples are rare: even our most up-to-date collection of all available NMPs only has the full text of NMPs before and after 2004 from four countries (Afghanistan, Colombia, Kenya, Uganda). The example of Kenya shows that legal rights or obligations appear in its 2008 NMP and not in its 1994 NMP. Conversely, both of Colombia’s 2003 and 2012 NMPs advocate for medicines as social goods, articulate health as a fundamental right, and promote measures to control medicines pricing.

### Implications for national pharmaceutical policy

Our basic hypothesis is that a NMP that explicitly identifies our 12 policy measures has the potential to address inequitable access to essential medicines by refocusing government policy and programmes on the medicines needs of the most vulnerable populations. Evaluating this hypothesis is difficult because few studies have examined the impacts of NMP provisions on medicines financing and affordability impacts on vulnerable populations. It is important to keep in mind that measuring the impact of a national policy is methodologically difficult as a control group is never possible. The next best methods are longitudinal time-series analyses, and observational studies which are often only post-intervention; but these methods do not allow for a reliable assessment of policy impact or causality. [24] One cross-sectional study of 64 NMPs by Holloway et al. identified a significant association between having a NMP (regardless of its content) and the provision of free medicines, a supply-side measure. [8] In another example, Colombia’s 2012–2021 National Pharmaceutical Policy promotes efficient spending on medicines through external reference pricing (among other measures), which was indeed implemented in 2013. [25,26] An impact study of the best selling 90 medicines suggests that this external reference pricing policy achieved a decrease in prices between 2011–2015, although sales and spending increased in the same period. [26] It is very likely that the external reference pricing system had an impact on the government’s capacity to finance medicines for vulnerable groups, but the causal relation cannot be proven.

Public financing and affordability of essential medicines for vulnerable groups has only been framed as a right in WHO’s NMP guidelines of 2001. Framing in health policy is gaining increasing attention as a means to reorient public health debates, the perception of problems, and their plausible solutions. [27] In our study, framing medicines as part of the right to health moves the debate away from *which* patients can afford health commodities, towards the question of *how* governments can provide basic essential goods to everyone, based on the common respect for the human dignity of all people. Human rights framing also implies certain obligations, such as the minimum core obligation of governments to provide essential medicines to those who can not provide for themselves. [16] Finally, a rights-based lens implies a certain set of principles towards achieving universal access to essential medicines, such as non-discrimination in medicines provision, maximising available government resources, seeking international assistance and cooperation, and selecting low-cost/most efficient policy options. [28] An example of human rights framing is found in the Philippines’ constitution, its 2011–2016 NMP, and national legislation for medicines. These documents all affirm the government’s duty to “make essential goods, health, and other social services available to all people at an affordable cost,” and guide national health insurance programmes for access to medicines. [29–31] Indeed, the 2011–2016 Philippine NMP and related national legislation for universal health coverage promotes government financing for essential medicines, efficient spending on medicines through cost-effectiveness measures, and guaranteed financial protection for vulnerable groups to access medicines. [10] Unfortunately, little data is available to concretely assess the ultimate impact of these measures on patient affordability and access.

The debate around indication-based pricing (i.e. different prices for different indications of the same medicine) is ongoing. From the human rights perspective, it is important that governments ensure medicines prices do not preclude access to essential medicines for patients. As with all governmental pricing policies, States should regularly monitor, evaluate, and report on the level of patient access, and remedy barriers when they occur.

We suggest that the innovative ideas and example texts identified in this article (Table 3 and online S2 Appendix) may form the basis of a balanced commitment to medicines affordability and financing in NMPs. We recommend that NMPs should address each of the 12 principles to find the right balance between the government’s duties as the primary funder of public sector pharmaceuticals, as the coordinator of all revenues (including user contributions and international funding), and as the steward of medicines selection, procurement and pricing.

### Implications for WHO policy

Updating WHO’s 2001 guidelines on NMPs and aligning them with WHO’s latest policies on essential medicines and human rights law will raise Member States’ awareness of the importance of human rights, their legal obligations, and the available policy measures to implement these duties in practice. Moreover, official WHO guidance on how to address UHC and embed the right to health in NMP text, with specific examples, can support ongoing national reforms or trigger other initiatives for universal access to essential medicines. Ultimately, enhanced legal commitments and political can catalyse inclusive progress towards universal access to essential medicines and the SDG for health.

How should WHO’s guidelines be revised? We recommend that WHO’s updated NMP guidelines should address critical gaps by explicitly referencing the State duty to provide essential medicines to those who need them, the participation of beneficiaries in medicines policy, and the creation of (non-judicial) accountability and redress mechanisms. If appropriately implemented, enhanced accountability and redress mechanisms, such as easy-to-access complaint and grievance procedures for patients, have the potential to swiftly remove access barriers. [32]

These mechanisms could also help stem the wave of spurious human-rights based litigation rising in some Latin American countries where many court cases claim access to publicly-funded, high-priced medicines using the misguided argument that the right to health entails immediate access to any treatment regardless of its price. [33–36] In previous work the authors have identified trends in UHC legislation for access to medicines. [10] We suggest that these same strategies could be embedded in NMPs to avoid deleterious medicine litigation; these trends are: articulating clear patient rights to access medicines proven to be cost-effective, and ensuring efficient and effective complaints procedures.

### Implications for research

Future research should investigate whether and how the commitments in NMPs are implemented in government practice. More investigation is also needed to determine the effectiveness of rights-based medicines policies at improving medicines affordability and equitable access for patients, and what the practical facilitators and barriers to implementation are.

### Strengths and limitations

Although our NMPs are sourced from the most comprehensive collection to date, our sample has more NMPs published before 2004, and from low-income countries. Retrieving few full-text NMPs from higher-income countries may be caused by governments self-reporting ‘yes’ in the WHO Pharmaceutical Sector questionnaire despite not having an official NMP (i.e. Mexico) or having a medicine law which functions as a NMP (i.e. Morocco). In some cases the full text of an official NMP was neither retrievable online nor through crowdsourcing in the E-drug network or targeted individual approaches.

We mitigated the risk of overlooking relevant policy content in our analysis by working with researchers fluent in the original language of the NMP and trained on the structure, standard terminology, and definitions used in the WHO guidelines and our checklist.

## Conclusion

Our study demonstrates to which extent a human rights-based approach to access to essential medicines within UHC schemes is integrated into 71 most recent NMPs, using a 12-point checklist focusing on medicines affordability and financing for vulnerable groups. Specific examples of how essential medicines and human rights principles are phrased in NMPs can be used by WHO and national policy makers to further align the goals and strategies of the national pharmaceutical sector with human rights law and the SDG targets for universal access to essential medicines.

## Supporting information

S1 Appendix Detailed methodology. Available at: https://figshare.com/s/f45dbb0b5556aae88855 (private link). DOI once published: 10.6084/m9.figshare.7008176.
(PDF)

S2 Appendix Examples of NMP texts. Available at: https://figshare.com/s/18f33754aad1c25d5c08 (private link). DOI once published: 10.6084/m9.figshare.7008191.
(PDF)
