# Supplementary material for: The right to health as the basis for universal health coverage: A cross-national analysis of national medicines policies of 71 countries
Source: PLoS One. 2019 Jun 28;14(6):e0215577. doi: 10.1371/journal.pone.0215577 (PMC6599146; doi:10.1371/journal.pone.0215577)
Supplement: S1 Appendix — Available at: https://figshare.com/s/f45dbb0b5556aae88855 (private link). DOI once published: 10.6084/m9.figshare.7008176. (PDF) [file pone.0215577.s001.pdf]

Detailed methodology for the project 'National medicines policy for universal access to medicines'

Original article: *The right to health as the basis for universal health coverage: A cross-national analysis of national medicines policies of 71 countries*

Authors: S. Katrina Perehudoff, Nikita V. Alexandrov, Hans V. Hogerzeil

Date: 16 July 2018

Introduction

This comparative study collects, describes, and assesses the text for access to medicines in national medicines policies (NMPs) from 71 countries against a 12-point normative framework and policy checklist of health systems and human rights principles.

WHO policy and legal framework

We identified overlapping principles in WHO's policies for essential medicines and international human rights law that are relevant for access to medicines, particularly their affordability and the financial protection of vulnerable groups.

We identified principles in the following WHO policies:

- Developing and Implementing a National Drug Policy, second edition (1),
- Equitable Access to Essential Medicines (2),
- Six Building Blocks of a Health System (3),
- Access to Medicines from a Health Systems Perspective (4),
- Good Governance for Medicines programme and Model Framework (5).

We selected corresponding principles from the following sources of international human rights law:

- International Covenant on Economic, Social and Cultural Rights (6),
- International Covenant on Civil and Political Rights (7),
- Optional Protocol on the International Covenant on Economic, Social and Cultural Rights (8),
- publications from the United Nations Committee on Economic, Social and Cultural Rights that interpret the scope and content of Covenant rights and obligations, including:
  - States duties under the Covenant (General Comment No. 3 (9)),
  - the right to health (General Comment No. 14 (10)),
  - authors' and inventors' rights in the context of access to medicines (General Comment No. 17 (11)),

- criteria to evaluate the reasonableness of State action in relation to available resources (Statement on the evaluation of the obligation to take steps "to a maximum of available resources" (12)),
- the right to social security (General Comment No. 19 (13)),
- the right to sexual and reproductive health, as part of the right to health (General Comment No. 22 (14)).

Principles for access to medicines in national law and policy

We identified overlapping principles for access to medicines through a multi-step, iterative process. The policy checklist was developed by two authors (KP and NVA) who shortlisted the relevant principles from source documents, independently piloted the shortlist on NMPs to determine their applicability and adequacy, and revised the shortlist. Three right to health and pharmaceutical policy experts (HVH, BT, E'tH) reviewed the shortlist to ensure the principles were sufficient and correctly defined.

Our final normative framework identifies 12 principles categorised in three domains (described below): legal rights and obligations, good governance, and technical implementation. The domains correspond to the structure-process-outcome framework for monitoring and evaluating the realisation of human rights by the UN Office of the High Commission for Human Rights. (15) Below we describe the principles assigned to each domain.

Legal rights and obligations

The 'Legal rights and obligations' domain reflects the essence of States' overarching right to health commitments that should be legally recognised in domestic law and policy. This domain consists of the individual entitlement to the highest attainable standard of health (**principle 1**) and the State's core obligation to provide essential medicines (**principle 2**).

These principles are primarily informed by the International Covenant on Economic, Social and Cultural Rights (ICESCR, art. 12) and the authoritative interpretation of essential medicines as a 'core obligation' of States in multiple General Comments. (6,9,10,13,14) To date, the only reference to human rights in the context of pharmaceutical policy is found in the Access to Medicines from a Health Systems Perspective framework proposed by Bigdeli and colleagues. (4) It includes a weak reference to human rights as a 'value'.

Good governance

The 'Good governance' domain captures principles to guide the processes of State action (i.e. *How* should States act?). This domain consists of transparency (**principle 3**), participation and consultation of beneficiaries (**principle 4**), monitoring and evaluation of State commitment, efforts and results (**principle 5**), and accountability and redress (**principle 6**).

From a legal perspective the International Covenant on Civil and Political Rights (ICCPR) offers a firm foundation to ground three of our four Good governance principles. (7,16) Transparency corresponds to the right to freedom of expression, including to seek, receive, and impart information (art. 19). (7,16) Participation relates to the right to take part in public affairs (art. 15). (7,16) Account-

ability is derived from the right to an effective remedy for rights violations (art. 2). (7,16) In addition, transparency, participation, and accountability emerge as common elements from the definitions of good governance provided by the International Development Association, the United Nations Development Programme, Office for the High Commissioner of Human Rights, former UN Commission on Human Rights, and other institutions (i.e. international financing institutions). (16) In a governance approach, monitoring is not an explicit principle because it is considered to be a component of accountability.

In addition, the Committee on Economic, Social and Cultural rights recognises in General Comment No. 14 that "good governance is essential to effective implementation of all human rights, including the realization of the right to health" (§55). (10) All four principles are reflected in General Comment No. 14 of the ICESCR (10). The participation of beneficiaries is required in relation to health-related decision making and processes for health policy, programming, and the organisation of health facilities goods and services (§11,17,54). (10) The monitoring and evaluation of State action for the realisation of health rights is a component of the right to health (§57-58). (10) Less specific are references to transparency and accountability in General Comment No. 14. A national health strategy and plan of action should be based on the principles of accountability and transparency (§43(f),55). (10) This concept creates a platform on which transparency and accountability can be related to State strategies and plans to realise the right to health.

From the perspective of WHO policy, we can look to the Good Governance for Medicines programme and Model Framework. (5) This framework advances 10 components (based on ethical principles) to guide laws, policies, and procedures to improve the management of and reduce corruption in pharmaceutical systems. Of the 10 components in this Model Framework, several corroborate our four Good governance principles. Transparency corresponds with the principle of 'transparent and accountable regulations and administrative internal and external audits' in which transparency is vaguely referenced (p.15). (5) Participation of beneficiaries is not strongly referenced in WHO's Good Governance for Medicines framework. It can be related to 'collaboration among anti-corruption and transparency initiatives' that includes civil society (p.15). (5) Monitoring is captured by the principle of 'management, coordination, and evaluation' (p.16). (5) Accountability is contained in the ethical principles 'accountable trusteeship' in which public servants are stewards of public resources and therefore accountable to the society they serve (p.12). (5)

Technical implementation

The 'Technical implementation' domain specifies the intermediate steps or policy measures that States should take to discharge their right to health obligations in the context of medicines (i.e. *What* should States do?). In previous research Perehudoff and Forman extensively examined how some of these principles are derived from international human rights law and WHO's policies. (17) They propose that 'reasonable' State action to provide essential medicines requires governments to:

- ensure sufficient public spending, which is at least the minimum amount required to purchase a basic package of essential medicines for all (**principle 8**),
- implement spending efficiencies through price control and the use of TRIPS Flexibilities when all other measures fail to yield affordable medicines (**principle 11**),

- generate efficiencies by seeking international (technical) cooperation and (financial) assistance to support domestic essential medicines programmes (**principle 10**);
- observe non-discrimination in national pharmaceutical policy through the financial protection of vulnerable groups, among other approaches (**principle 12**).

In addition, two other principles arose from overlapping concepts in WHO's policies and international human rights law: the selection of essential medicines (**principle 7**) and the pooling of user contributions to increase the resources available for pharmaceuticals (**principle 9**).

Methods

Data collection

Between January to October 2015 NVA conducted a systematic search to identify (i.e. by title, number, or date of publication, and/or full-text documents) all retrievable NMPs for each of WHO's 195 Member States. This procedure included searching:

- online repositories (i.e. WHO Essential Medicines and Health Products Information Portal; WHO Pharmaceutical Country Profiles 2003, 2007, and 2013);
- government websites (i.e. national Ministries of Health and other health agencies);
- Google search engine using the term (in English, Dutch, Spanish, French, or Russian, as relevant) national medicine(s)/drug(s)/pharmaceutical policy + name of country + year of publication (if known)

We used academic literature to cross-reference the search results and locate other NMPs. We further expanded our search through a global call for NMPs from targeted countries through the E-DRUG online network.

We included one official NMP per country. We excluded draft, incomplete, and unclear medicine policy documents, policies addressing a specific component (i.e. intellectual property management), and documents in other languages besides English, Dutch, French, or Spanish. Our search method, previously reported in Wirtz et al., yielded 67 full text NMPs, which were deposited in WHO's online Essential Medicines and Health Products Information Portal (publicly accessible here: http://apps.who.int/medicinedocs/en/). Between January 2017 to March 2018 we received 13 additional full text NMPs that met the inclusion criteria.

Collection of legislation

We included NMP texts (i.e. legal provisions) in our analysis by extracting legal texts that correspond to our 12 principles using a keyword search, followed by a manual search to catch omissions. Key words were related to the 12 principles; they included: medicines, pharmaceutical, drugs, medications, right, entitle, oblige/obligation, responsible/responsibility, guarantee, must, transparency, participate/participation, consultation, monitor, accountability, complain, selection, consumer, price, compulsory, generic, access, international/foreign, donor, vulnerable, poor, and indigent.

Applying the normative framework

Our normative framework serves as both a policy checklist for assessing national policy, and a 'wish list' to guide policy reform.

We calculated the reliability of NMP text selection by two coders using Cohen's Kappa. KP and NVA each independently coded six randomly selected countries (approx. 8% of the sample countries: Botswana, Ethiopia, Fiji, Malawi, Oman, Timor-Leste). We extracted the same NMP texts in 75.7% of cases with a Cohen's K=0.695, which indicates that 69.5% of similarities between coders were not due to chance (0.61<Cohen's K<0.8 suggests 'substantial' agreement).

Given the substantial agreement between coders, two authors (KP and NVA) worked with one half of the NMPs each to independently graded the strength of each principle in the policy texts on a three-point coding matrix (see Table 1 below). Where possible, strength was determined using the human rights concepts of State commitments (i.e. dedication to realising rights) and State actions (i.e. efforts or steps to achieve a goal). In some cases principles could not be judged in terms of State commitments and State action in the legal texts; therefore, the strength of the legal texts was determined by the clarity of the policy commitments. The three points were generally defined as follows and then tailored to the policy text:

- A strong text recognises the principle and adopts mechanisms to implement it (i.e. codifies the State commitment and State action). If the text cannot be judged in this way, then a strong text refers to a clear commitment to medicines affordability and/or financing;
- A weak text recognises either a vague State commitment or (a single) action;
- No relevant text could be found in the legislation.

The coding matrix is found in Table 1 below.

All codes and source text were independently reviewed (by both KP and NVA) who discussed inconsistencies and jointly agreed on the final codes.

Data analysis

We report the frequency of each principle in NMPs and describe the different approaches in different countries.

We hypothesised that the content of WHO's 2001 NMP guidelines would inform the content of subsequent NMPs. Therefore we divided NMPs between those adopted in or before 2003 (n=32) and those adopted in or after 2004 (n=39). Associations were determined in SPSS version 25 using Pearson's Chi-squared statistic with significance set at $p<0.05$.

Table 1. Assessment tool for access to essential medicines in national law and policy.

Principles	Original human rights principle	WHO essential medicines policy	Coding matrix
**Legal rights and obligations**			
1. Right to health	Right to the highest attainable standard of health	Human rights are a 'value'. (Bigdeli et al. 2013)	Black = Clear endorsement of the right to health of all; may be related to medicines.<br>Grey = Vague reference to the right to health or rights of patients, consumers, or users.<br>White = No entitlement.
2. State obligation to provide essential medicines	Core obligation to provide essential medicines defined by WHO		Black = Absolute State obligation to ensure/guarantee access to (essential) medicines for all or to take measures so everyone can access the medicines they need.<br>Grey = Vague State duty to provide healthcare or implement the NMP, or a shared duty between the State and others to provide medicines.<br>White = No obligations .
**Good governance**			
3. Transparency	Transparency	Includes information to assess service access and coverage, and publicly available price information for medicines. A component of good governance for medicines. (Hodgkin *et al.*, 2001; WHO, 2007, 2014)	Black = Transparency measures in relation to medicines affordability and financing.<br>Grey = Transparency measures in general.<br>White = No transparency measures.
4. Participation & consultation	Participation	Collaboration and accountability of all health systems actors, and stakeholder consultation is required. Referenced in good governance for medicines. (Hodgkin *et al.*, 2001; WHO, 2007, 2014)	Black = Participation and consultation measures in relation to medicines affordability and financing.<br>Grey = Participation and consultation measures in general.<br>White = No participation and consultation measures.
5. Monitoring & evaluation	Monitoring	Achieved through explicit government commitment, indicator-based surveys, and independent impact evaluation. A component of good governance for medicines. (Hodgkin *et al.*, 2001; WHO, 2004, 2007, 2014)	Black = Monitoring and evaluation measures for medicines affordability and financing.<br>Grey = Monitoring and evaluation measures in general.<br>White = No monitoring or evaluation measures.

6. Accountability & redress	Accountability	Accountability of all health systems actors. (WHO, 2007)	Black = Accountability and redress measures if an individual is unable to access the medicine he/she requires.<br>Grey = Accountability in general is acknowledged.<br>White = No recognition of accountability nor redress.
**Technical implementation**			
7. Selection of essential medicines	(Assured) quality of health services (of the AAAQ)	Includes the essential drugs concept, procedures to define and update the national list(s) of essential drugs, explicit, evidence-based criteria that includes cost-effectiveness, and selection mechanisms. (Hodgkin *et al.*, 2001; WHO, 2004)	Black = Comprehensive approach (principle of medicines selection AND mechanisms for selection)<br>Grey = Vague principle OR a single policy measure without a comprehensive approach to essential medicines.<br>White = No recognition of essential medicines.
8. Government financing	Duty to adopt appropriate legislative, administrative, budgetary and other measures to a maximum of its available resources.<br><br>Core obligation to provide essential medicines as defined by WHO	Requires adequate funding and mobilising all available public resources and increase funding for priority diseases, and the vulnerable. (Hodgkin *et al.*, 2001; WHO, 2004, 2007)	Black = Clear State obligation to finance (essential) medicines and a specific policy measure.<br>Grey = Vague State commitment (i.e. to increase budget for medicines) or shared responsibility of State and others.<br>White = No government financing.
9. Pool user contributions		Medicines reimbursement with user charges is a (temporary) financing option. (WHO, 2004, 2007)	Black = Provision of primary care medicines free-of-charge/for nominal fee, co-payments for other medicines, and exceptions for those who can not pay.<br>Grey = Principle of cost recovery, reimbursement, or joint responsibility of State and users to finance medicines.<br>White = No concept of nor criteria for user contributions.
10. International assistance and technical co-operation	Duty to seek international assistance and technical cooperation	Includes the possibility of using development loans for medicines financing. (WHO, 2004)	Black = Financial aid or/and technical assistance from the international community (not only the private sector).<br>Grey = Reference to international cooperation for health/UHC.<br>White = No means for international co-operation.

11. Efficient and cost-effective spending	Duty for the efficient use of available resources<br>Duty to take appropriate steps to ensure that the private business sector is aware of, and consider the importance of, the right to health in pursuing their activities.<br>Duty to prevent unreasonably high costs for access to essential medicines from undermining the rights of large segments of the population to health.<br>Duty to seek low-cost policy options.	Includes the efficient use of resources and affordable pricing through: price control; a pricing policy for all medicines; competition through generic policies and substitution; good procurement practices; price negotiation and information; and TRIPS-compliant measures such as compulsory licensing and parallel imports. (Hodgkin *et al.*, 2001; WHO, 2004, 2007)	Black = Principle of cost-effectiveness / efficiency, AND one or more mechanisms in relation to medicines.<br>Grey = Either the principle OR mechanisms for cost-effectiveness/efficiency, but not both. More generally about health care/UHC.<br>White = No principle and mechanisms for spending.
12. Financial protection of vulnerable groups	Duty towards non-discrimination and attention to the vulnerable	Increase government funding for poor and vulnerable groups and reduce the risk of catastrophic health spending. (Hodgkin *et al.*, 2001; WHO, 2004)	Black = Clear State duty to finance UHC package / essential medicines for all vulnerable people.<br>Grey = Vague State duty (i.e. exemption for some vulnerable people but unclear whether State finances their medicines)<br>White = No financial coverage of the poor .

Acknowledgements: We thank Prof. Brigit Toebes (BT) and Dr. Ellen 't Hoen (EtH) for reviewing our assessment tool, as well as Ms. Femke Bloem and Mr. José Castela Forte for their research assistance.

References

1. Hodgkin C, Carandang ED, Fresle DA, Hogerzeil HV, editors. How to develop and implement a national drug policy [Internet]. Second edition. Geneva: World Health Organization; 2001 [cited 2018 May 18]. Available from: http://apps.who.int/iris/bitstream/handle/10665/42423/924154547X.pdf?sequence=1
2. World Health Organization. Equitable access to essential medicines: a framework for collective action [Internet]. Geneva: World Health Organization; 2004 [cited 2018 May 23]. 6 p. Available from: http://apps.who.int/medicinedocs/pdf/s4962e/s4962e.pdf
3. World Health Organization. Everybody's business- strengthening health systems to improve health outcomes [Internet]. Geneva: World Health Organization; 2007 [cited 2018 May 18]. 44 p. Available from: http://apps.who.int/iris/bitstream/handle/
4. Bigdeli M, Jacobs B, Tomson G, Laing R, Ghaffar A, Dujardin B, et al. Access to medicines from a health system perspective [Internet]. Health Policy Plan. 2013 [cited 2018 May 22];28(7):692–704. Available from: https://academic.oup.com/heapol/article/28/7/692/819804
5. World Health Organization. Good governance for medicines model framework [Internet]. Geneva: World Health Organization; 2014 [cited 2018 May 31]. 54 p. Available from: http://apps.who.int/iris/bitstream/handle/10665/129495/9789241507516_eng.pdf?sequence=1
6. UN General Assembly. International Covenant on Economic, Social and Cultural Rights [Internet]. 1966 [cited 2018 May 23]. Adopted in Resolution No. 2200A (XXI). Available from: http://www.ohchr.org/EN/ProfessionalInterest/Pages/CESCR.aspx
7. UN General Assembly. International Covenant on Civil and Political Rights [Internet]. 1966 [cited 2018 May 31]. Available from: http://www.ohchr.org/EN/ProfessionalInterest/Pages/CCPR.aspx
8. UN General Assembly. Optional Protocol of the Covenant on Economic, Social and Cultural Rights [Internet]. 2008 [cited 2018 May 31]. Adopted in Resolution No.: A/RES/63/117. Available from: http://www.ohchr.org/EN/ProfessionalInterest/Pages/OPCESCR.aspx
9. UN Committee on Economic, Social and Cultural Rights. General Comment No. 3 on The Nature of States Parties' Obligations [Internet]. 1991 [cited 2018 May 23]. Document No.: E/1991/23. Available from: http://tbinternet.ohchr.org/_layouts/treatybodyexternal/Download.aspx?symbolno=INT%2fCESCR%2fGEC%2f4758&Lang=en
10. UN Committee on Economic, Social and Cultural Rights. General Comment No. 14 on the Right to the Highest Attainable Standard of Health [Internet]. 2000 [cited 2018 May 23]. Document No.: E/C.12/2000/4. Available from: http://tbinternet.ohchr.org/_layouts/treatybodyexternal/Download.aspx?symbolno=E%2fC.12%2f2000%2f4&Lang=en
11. UN Committee on Economic, Social and Cultural Rights. General Comment No. 17 on The Right of Everyone to Benefit from the Protection of the Moral and Material Interests Resulting from any Scientific, Literary or Artistic Production of Which He or She is the Author [Internet]. 2006 [cited 2018 May 31]. Document No.: E/C.12/GC/17. Available from: http://tbinternet.ohchr.org/_layouts/treatybodyexternal/Download.aspx?symbolno=E%2fC.12%2fGC%2f17&Lang=en
12. UN Committee on Economic Social & Cultural Rights. An evaluation of the obligation to take steps to the "maximum of available resources" under an Optional Protocol to the Covenant [Internet]. 2007 [cited 2018 May 31]. Document No.: E/C.12/2007/1. Available from: http://www2.ohchr.org/english/bodies/cescr/docs/statements/Obligationtotakesteps-2007.pdf
13. UN Committee on Economic, Social and Cultural Rights. General Comment No. 19 on the Right to Social Security [Internet]. 2008 [cited 2018 May 31]. Document No.: E/C.12/GC/19. Available from: http://tbinternet.ohchr.org/_layouts/treatybodyexternal/Download.aspx?symbolno=E%2FC.12%2FGC%2F19&Lang=en
14. UN Committee on Economic, Social and Cultural Rights. General Comment No. 22 on the Right to Sexual and Reproductive Health [Internet]. 2016 [cited 2018 May 23]. Document No.: E/C.12/GC/22.

Available from: http://tbinternet.ohchr.org/_layouts/treatybodyexternal/Download.aspx?symbolno=E%2fC.12%2fGC%2f22&Lang=en

15. Office of the High Commissioner for Human Rights. Human rights indicators: a guide to measurement and implementation [Internet]. 2012 [cited 2018 May 18]. Document No.: HR/PUB/12/5. Available from: http://www.ohchr.org/Documents/Publications/Human_rights_indicators_en.pdf

16. Lane L. A proposal to move beyond achieving horizontal effect of human rights through international human rights law. In: The horizontal effect of international human rights law: Towards a multi-level governance approach. Groningen: University of Groningen; 2018. 317-432.

17. Perehudoff SK, Forman L. What constitutes 'reasonable' State action on core obligations? Considering a right to health framework to provide essential medicines. Submitted.

18. Wirtz VJ, Hogerzeil H V, Gray AL, Bigdeli M, De Joncheere CP, Ewen MA, et al. Essential medicines for universal health coverage. Lancet [Internet]. 2016 [cited 2018 May 18];1-74. Available from: http://apps.who.int/medicinedocs/documents/s23079en/s23079en.pdf
