# Supplementary material for: The right to health as the basis for universal health coverage: A cross-national analysis of national medicines policies of 71 countries
Source: PLoS One. 2019 Jun 28;14(6):e0215577. doi: 10.1371/journal.pone.0215577 (PMC6599146; doi:10.1371/journal.pone.0215577)
Supplement: S2 Appendix — Available at: https://figshare.com/s/18f33754aad1c25d5c08 (private link). DOI once published: 10.6084/m9.figshare.7008191. (PDF) [file pone.0215577.s002.pdf]

# Examples of legal text from the project 'National medicines policy for universal access to medicines'

Original article: *The right to health as the basis for universal health coverage: A cross-national analysis of national medicines policies of 71 countries*

Authors: S. Katrina Perehudoff, Nikita V. Alexandrov, Hans V. Hogerzeil

Date: 16 June 2018

This Annex presents an overview of innovative text for access to medicines identified in national medicines policies (listed in Table 3 of the article). The legal texts below have been edited for clarity.

| **1. Right to health including essential medicines** |
|---|
| **El Salvador (2011)**: The population has the right to have safe, effective and safe medicines of quality in a legal framework of equity, social justice and solidarity.<br>**Southern Sudan (2006)**: The vision of the Government of Southern Sudan is to pursue the ideals of the right to health and access to essential medicines so that everyone may attain the full benefit of quality of life, which will result in the economic development and prosperity of the nation. |
| **2. State duty to provide pharmaceuticals** |
| **Indonesia (2006)**: The government is responsible for the availability, affordability and fair distribution of essential medicines in order to fulfil the needs of the public.<br>**Iran (2004)**: The prime responsibility of the Ministry of Health & Medical Education is to ensure consumers' access to those drugs included in the "Iranian National Formulary". The government will ensure that pharmaceutical benefits are provided to all Iranian citizens through universal health insurance coverage.<br>**Philippines (2011-2016)**: The State plays the primary role in the progressive realization of equitable access to medicines for all its citizens, especially the poor. Filipinos shall not be denied access nor become impoverished because of high drug costs.<br>The national and local governments shall ensure that essential health packages are available in public health facilities.<br>**Uganda (2015)**: The government is required to work towards the progressive realization of the highest attainable standard of health, expanding health services to progressively achieve UHC with essential services, using a primary health care approach. |
| **3. Transparency of governments' action and outcomes for medicines affordability** |
| **Iran (2004)**: It is a basic right of medical providers, and patients as well, to have access to valid and well-documented information through national drug information centers.<br>**Philippines (2011-2016)**: The government shall undertake these measures for medicines pricing: [Introduce an] electronic essential drug price monitoring system and drug price reference index to foster transparency and |

| |
|---|
| accountability in pricing and monitor the trends and the impacts of government policies and interventions. |
| **4. Participation and consultation for medicines affordability** |
| **New Zealand (2007)**: Stakeholders (including consumers) understand and have the opportunity, as appropriate, to participate in the decision-making process used for regulating, funding and managing medicines. |
| **5. Monitoring and evaluation for medicines affordability** |
| **Colombia (2012)**: Market monitoring requires a toolbox to surveil, detect, resolve, and regulate market distortions by : (c) identifying reasons for the low use of TRIPS Flexibilities in Colombia and, if appropriate, developing the supportive regulatory frameworks; (d) studying whether the intellectual property system in Colombia has influence on 'drug prices' and establishing a public system for periodic monitoring to track this information.These objectives fall to the Monitoring Committee, which is also responsible for establishing a mechanism to monitor compliance with the universal provision of essential medicines services, with an emphasis on priority diseases.<br><br>**Philippines (2011-2016)**: Drug prices shall be actively monitored by the Department of Health enabling transparent and objective price information sharing with drug procuring entities, consumers, health professionals and the public.<br><br>**Tajikistan (2003)**: To introduce effective strategies aimed at the improvement of the pharmaceutical sector of the country, monitoring and assessing the priorities of the National Drug Policy and its implementation will be conducted. In the assessment auxiliary indicators (population, economic data, human resources, health protection system data etc.), structural qualitative indicators (legislation and regulation, selection of essential drugs and their registration, drug financing, mechanisms of drug supplies, pricing policy, medical information and education etc.) and quantitative indicators of the process and result (availability and accessibility of essential drugs, quality, efficient utilization of drugs etc.) will be used. |
| **6. Accountability and redress for medicines affordability** |
| **Afghanistan (2014)**: Accountability and transparency [are promoted] through clearly defined responsibilities and open procedures and systems. It seeks to be responsive and inclusive by defining a role for the patients and customers, and formalizing complaints procedures and appeals.<br><br>**Kenya (2008)**: Relevant and unbiased information will be made available to enable consumers to use prescribed and 'over the counter' medicines in order to maximize the therapeutic benefits and minimize any associated risks. Establish an effective mechanism for consumer feedback and complaints on medicines issues.<br><br>**Malaysia (2012)**: [Under the heading 'medicines quality'] All complaints pertaining to medicines shall be investigated and appropriate action shall be taken in a timely manner. |
| **7. Selection of essential medicines** |
| **Philippines (2011-2016)**: The Department of Health shall define the essential health package or minimum provision of essential medicines at different levels of the health care system and the government should ensure their availability in public health facilities.<br><br>Essential drug packages, once declared as entitlements by government, shall be provided for free or with reasonable co-payments for patients either through direct subsidy by the Department of Health or social health insurance and any other payment schemes of government.<br><br>**South Africa (1996)**: Drug selection: This aim will be achieved through the development of an Essential Drugs Programme, which will include an Essential Drugs List and standard treatment guidelines.<br><br>A National Essential Drugs List Committee (NEDLC), appointed by the Minister of Health, will be responsible for the |

selection of drugs to be used in the public sector. The Committee will be composed of experts in all spheres of medical and pharmaceutical practice, including clinical pharmacists and pharmacologists, medical specialists, a paediatrician, professional nurses from community practice, medical practitioners involved in primary care practice, a member of a drug information centre, a member of the clinical committee of the Medicines Control Council, a health professional involved in drug management training and representatives of the provincial Essential Drugs List committees. Additional members may be co-opted on an ad-hoc basis. Consultations will be undertaken with all interested parties.

The NEDLC will draw up and periodically review a National List of Essential Drugs using generic names. This list will be prepared for three levels of health care providers, namely primary contact, secondary and tertiary hospital care. The list will be reviewed every two years. It will be distributed to all health workers in the country.

The selection of drugs on the National Essential Drugs List will be based on the following criteria: must meet the health needs of the majority of the population; sufficient proven scientific data must be available regarding the effectiveness of any such product; products should have a substantial safety and risk/benefit ratio; the aim, as a general rule will be to include, as far as possible, only products containing single pharmacologically active ingredients. Combination products may, as an exception, be included where patient compliance becomes an important factor or two pharmacologically active ingredients are synergistically active in a product when two or more drugs are equivalent in the above respects, preference will be given to those which: have the best cost advantage, have best pharmacokinetic properties, has been researched the most, have the best patient compliance, and have the most reliable local manufacturer.

The National List of Essential Drugs will be used as a foundation for: the basic health care package of the National Health System for Universal Primary Care; procurement and use of drugs, standard treatment guidelines and training in rational prescribing, drug information to health care providers including a national formulary, support to the national pharmaceutical industry, drug donations. The list may also be used as a model for medical aid schemes.

**8. Sufficient government financing for essential medicines**

**Afghanistan (2014)**: It is the Ministry of Public Health's responsibility as defined in the National Health Policy and Strategy to ensure stable and adequate financing for health care as a whole despite increasing challenges. The Ministry must also ensure that the financing of medicines supply is fairly shared between the Government and consumers and that stringent price control is maintained and wastage reduced. At all times, the Ministry must ensure that spending is in line with priorities, that there is sufficient transparency in the allocation of financial resources, that the various sources of funding are coordinated, and that the different mechanisms for financing the delivery of health services are monitored for cost-efficiency and acceptability.

**Nigeria (2005)**: Financing the various provisions of the National Drug Policy shall be the primary responsibility of government at all levels. Participation in the National Health Insurance Scheme by individuals, organisations and communities shall, however, be encouraged. In order to realise the objectives of the National Drug Policy, the Federal Government shall ensure that:

Suitable financial provisions are made within the total health budget for sustainable implementation and monitoring of the policy;

Adequate budgetary allocations are made for drugs, in line with internationally recommended norms;

Priority is given to the provision of adequate funds for drugs used in primary health care and the control of endemic diseases;

The cost of drugs to the patient is low, but sufficient to recover total costs with a little mark-up for administrative expenses and adequate maintenance of the drug revolving fund;

The costs of the promotional and preventive aspects of the National Drug Policy like health information and education, human resources development and research are fully borne by government;

| |
|---|
| Government at all levels make specific budgetary provisions to cover the cost of exemptions which shall apply to such categories of patients as accident victims, tuberculosis patients, the destitute, the mentally retarded, children, and the elderly. |
| **9. Pooling user contributions for essential medicines** |
| **Eritrea (2010)**: The government will ensure that essential medicines are available to all people in need. To this end, medicines will be provided at a nominal price at the point of service at the primary care level. All medicines at the primary care level will be provided at a nominal price. At the secondary and tertiary levels a fixed affordable co-payment for medicines supplied by the State will be levied. A system of exemption will be established for patients without the resources to meet such payment to ensure they are not deprived of treatment. |
| **10. International assistance and technical cooperations for medicines affordability** |
| **Ecuador (2007)**: In the context of long-term objectives on medicines pricing, the government:<br>Promotes, jointly with other countries, the conclusion of reference price policies to acquire essential drugs, orphans and exclusive medicines; as well as those used for the treatment of catastrophic and high-cost diseases;<br>Designs and implements an information system with international organizations of health and other countries to know referential prices, suppliers of medicines, prices of raw materials and active ingredients that are marketed, to modernize and make transparent the pricing system for medicines for human use.<br>**Ghana (2004)**: In the context of medicines financing, the NMP commits:<br>To establish a system that ensures joint responsibility between government and consumers for drug financing, which will also provide for the vulnerable section of the population. The government shall collaborate with the private sector and donor agencies in the funding of drug supplies to the public sector;<br>To provide the needed drugs to adequately treat and control [emerging] diseases, and also make other resources available where there are special needs. The Ministry of Health shall collaborate with the relevant international bodies to mobilise resources for these cases, where they cannot be provided from the country. |
| **11. Efficient and cost-effective spending on essential medicines** |
| **Ecuador (2007)**:<br><u>Accessibility</u>:<br>Declare that public health and access to medicines are about patents. The State reserves the right to grant compulsory licenses and / or import parallel operations; especially in cases of catastrophic illnesses and emergencies.<br>Promote the joint participation of public and private institutions and non-governmental organizations, in the processes of joint acquisition of drugs, at the best price.<br>Ensure the availability and accessibility of the population to generic medicines, for the treatment of the most prevalent diseases; with emphasis on the selection and rational use, affordable prices, financial sustainability and reliable supply systems.<br>Stimulate the production of generic drugs by pharmaceutical laboratories and promote foreign investment, to expand the supply in the Ecuadorian market.<br>Promote the prescription of generic medicines- of greater therapeutic value for prescribing professionals- in order to reduce health spending and ensure its cost-effective use, at all levels of the health system, both public as private.<br>Monitor that the prescription of medicines, both in the public and private sectors, contains the generic description of the drug to allow patients to choose the equivalent product according to the availability of their resources. |

<u>Registration</u>:

Authorize compulsory licensing and parallel imports of medicines, for public health reasons deemed necessary, which can facilitate the rapid commercialisation of generic medicines.

<u>Pricing</u>:

a. Short term

- The pricing process must be done according to the target population. This is to approach the problem from the point of view of the population; and, not from the point of view of the industry.
- Maintain price control, improving the system of fixing them with attachment unrestricted by legislation, by industry and the State, through the Council of Pricing of Medicines for Human Use.
- Establish a clear division of responsibilities, between the Technical Commission, as advisory body; and, the Council, as manager.

b. Medium term

- Segment the products of the pharmaceutical market to implement a differentiated pricing policy, which encourages the generic market of the country, and, control the exclusive products. In the case of products that fall outside the pricing scheme, the laboratories or distributors should report their prices to the Ministry of Public Health. The prices set, together with the marketing prices reported by the laboratories or distributors, should be consolidated into a price list, which should be sent to all health institutions.
- Establish monitoring and penalization mechanisms for those who do not observe the list of prices.

c. Long term, Establish additional guidelines and strategies:

- Implement a policy of controlled price release in the market for non-exclusive products, which have no less than four competitors from unrelated (legal or natural) persons.
- Ensure the participation of the Ministries of Health, Economy and Industry in the design of the mechanisms that allow to reach an adequate economic regulation.
- Develop the free supply and demand of medicines, preserving their quality, with effectiveness and safety; through the implementation of a "Differentiated Pricing Policy", that encourages the generic market in the country and control exclusive products.
- Regulate the price of marketed medicines according to indicators of national and international market, economic conditions, purchasing power of users, until the competition and the laws of supply and demand, regulate the pharmaceutical market, prevailing the interests of the population.
- Promote, jointly with other countries, the conclusion of reference price policies to acquire essential drugs, orphans and exclusive medicines; as well as those used for the treatment of catastrophic and high-cost diseases.
- Design and implement an information system with international health organizations and other countries to know referential prices, suppliers of medicines, prices of raw materials and active ingredients that are marketed, to modernize and make transparent the pricing system for medicines for human use.
- To reach a consensus among the actors of the National Health System on the alternative most viable approach to the Price Control System.
- Carry out the technical studies necessary to establish the parameters for define which products require pricing or not.

| 12. Financial protection of the poor and vulnerable |
| --- |
| **Jordan (2014)**: Government at all levels makes specific budgetary provisions to cover the cost of exemptions which shall apply to such categories of patients as poor, mentally retarded [sic], children, and the elderly, etc.<br>**Philippines (2011-2016)**: The Department of Health, Philhealth and other relevant government agencies shall employ strategies that will provide free medicines to the poor or a population of patients that addresses priority diseases (e.g. tuberculosis, HIV, malaria, cancers). Where applicable, medicines shall be provided for free especially in primary healthcare facilities.<br>**Timor Leste (2010)**: An important objective is to strive towards equity and efficiency in access to medicines of reliable quality for all citizens and/or visiting people in country, regardless of social vulnerability, poverty or any form of social marginalization. Special funding provisions shall be made for the low-income and especially vulnerable groups of the population who are unable to pay for their treatment. |
